# Cemeteries in Miami-Dade County, Florida are important areas to be targeted in mosquito management and control efforts

**DOI:** 10.1371/journal.pone.0230748

**Published:** 2020-03-24

**Authors:** André B. B. Wilke, Chalmers Vasquez, Augusto Carvajal, Maday Moreno, Yadira Diaz, Teresa Belledent, Laurin Gibson, William D. Petrie, Douglas O. Fuller, John C. Beier

**Affiliations:** 1 Department of Public Health Sciences, Miller School of Medicine, University of Miami, Miami, FL, United States of America; 2 Miami-Dade County Mosquito Control Division, Miami, FL, United States of America; 3 Department of Geography and Regional Studies, University of Miami, Coral Gables, FL, United States of America; University of Crete, GREECE

## Abstract

Definable habitats at the neighborhood level provide a wide range of favorable habitats with optimal conditions and environmental resources for mosquito survival. Problematic habitats for controlling mosquitoes in urban environments such as tire shops, bromeliad patches, and construction sites must be taken into consideration in the development of effective mosquito management and control in urban areas. Cemeteries are often located in highly urbanized areas serving as a haven for populations of vector mosquito species due to the availability of natural resources present in most cemeteries. Even though Miami-Dade County, Florida was the most affected area in the United States during the Zika virus outbreak in 2016 and is currently under a mosquito-borne illness alert after 14 confirmed locally transmitted dengue cases, the role of cemeteries in the proliferation of vector mosquitoes is unknown. Therefore, our objective was to use a cross-sectional experimental design to survey twelve cemeteries across Miami-Dade County to assess if vector mosquitoes in Miami can be found in these areas. Our results are indicating that vector mosquitoes are able to successfully exploit the resources available in the cemeteries. *Culex quinquefasciatus* was the most abundant species but it was neither as frequent nor present in its immature form as *Aedes aegypti* and *Aedes albopictus*. This study revealed that vector mosquitoes, such as *Ae*. *aegypti*, *Ae*. *albopictus*, and *Cx*. *quinquefasciatus* are successfully exploiting the resources available in these areas being able to thrive and reach high numbers. Mosquito control strategies should consider both long-term strategies, based on changing human behavior to reduce the availability of aquatic habitats for vector mosquitoes; as well as short-term strategies such as drilling holes or adding larvicide to the flower vases. Simple practices would greatly help improve the effectiveness of mosquito management and control in these problematic urban habitats.

## Introduction

Vector mosquitoes such as *Aedes aegypti*, *Aedes albopictus*, and *Culex quinquefasciatus* are expanding their range globally [1–3]. Invasive mosquito vector species often benefit from the decrease in the overall biodiversity of species due to an increase in urbanization [4]. Such biotic homogenization processes not only increase the contact between human hosts and mosquito vectors but also act as drivers that positively increase the range and abundance of populations of mosquito vector species [4–7].

The increase in range and abundance of vector mosquitoes can be deemed as one of the factors for the substantial increase in the incidence of vector-borne diseases (VBDs) [8,9]. In 2019 alone, 2,853,248 million cases of dengue have been reported in the Americas [10], and the current estimates for the global human dengue infections range around 390 million cases per year with no signs of slowing down [11]. Moreover, VBD outbreaks are not only being reported more frequently in endemic areas [10,12,13], but are also been reported in formerly non-endemic countries such as Croatia, France, and Italy [14–16].

There are only a few vaccines and treatments available to prevent arbovirus infections. However, they are not enough to control and prevent VBD outbreaks. Despite the availability of a safe and effective yellow fever vaccine, many logistic issues with its production and distribution as well as public adherence to vaccination campaigns have impaired its effectiveness [17–20]. Recent major outbreaks in South America caused hundreds of deaths [21]. The situation in Africa is even more troublesome, yellow fever virus outbreaks in Angola and in the Democratic Republic of the Congo have resulted in thousands of deaths and current death estimates in the entire continent range around 78,000 deaths per year [22,23].

Considering the above scenario, controlling vector mosquito populations is the most effective way to prevent the transmission of VBDs [24], and has been proven feasible under the Integrated Vector Management framework (IVM) [25]. Controlling vector mosquitoes is a complex and dynamic task that relies on many actions that logically add to each other [26]. Effective surveillance is an essential part of IVM, and the development of effective mosquito management and control relies on the behavior of the target species and where they are concentrated, abundant, and harder to reach.

Definable habitats at the neighborhood level provide a wide range of favorable habitats with optimal conditions and environmental resources for mosquito survival. Problematic habitats for controlling mosquitoes in urban environments such as tire shops [27], bromeliad patches [28], and construction sites [29] must be taken into consideration in the development of effective mosquito management and control in urban areas.

IVM is currently the gold standard for controlling vector mosquitoes [26]. It has achieved success in the past in eliminating vector mosquitoes from urban areas [30]. However, the implementation of mosquito management and control operations based on the IVM is complex and requires multiple approaches in an interdisciplinary framework that builds on each other in a systematic manner. Moreover, the lack of evidence of the effectiveness of new tools to control vector mosquitoes based on genetically modified mosquitoes added to their prohibitive cost put these approached far from being used in real-world conditions [31]. Therefore, understanding how vector mosquitoes are exploiting the resources available in urban habitats and how they are concentrated in urban areas is key for the identification of modifiable urban features to guide and improve the development of effective mosquito management and control strategies in urban areas.

In this context, the role of cemeteries in the proliferation of vector mosquitoes and in monitoring VBD transmission has been intensively studied over the years. [32–38]. Cemeteries are often located in highly urbanized areas serving as a haven for populations of vector mosquito species due to the availability of natural resources present in most cemeteries such as: (i) sugar sources, from both the natural vegetation and ornamental flowers; (ii) widely available vegetated areas for mosquitoes to rest; (iii) high availability of breeding habitats in the form of flower vases, ornamental bromeliads, and adornment items such as toys and fishbowls; and (iv) large presence of human hosts for blood-feeding.

Even though Miami-Dade County, Florida was the most affected area in the continental United States during the Zika virus outbreak in 2016 [39], 212 imported dengue cases were reported in 2019 [40] and Miami is currently under a mosquito-borne illnesses alert after 14 confirmed locally transmitted dengue cases [41]. The role of cemeteries in the proliferation of vector mosquitoes in Miami is unknown. Therefore, our objective was to use a cross-sectional experimental design to survey twelve cemeteries across Miami-Dade County to assess if vector mosquitoes can be found in these areas.

## Methods

### Study design

**Fig 1:**
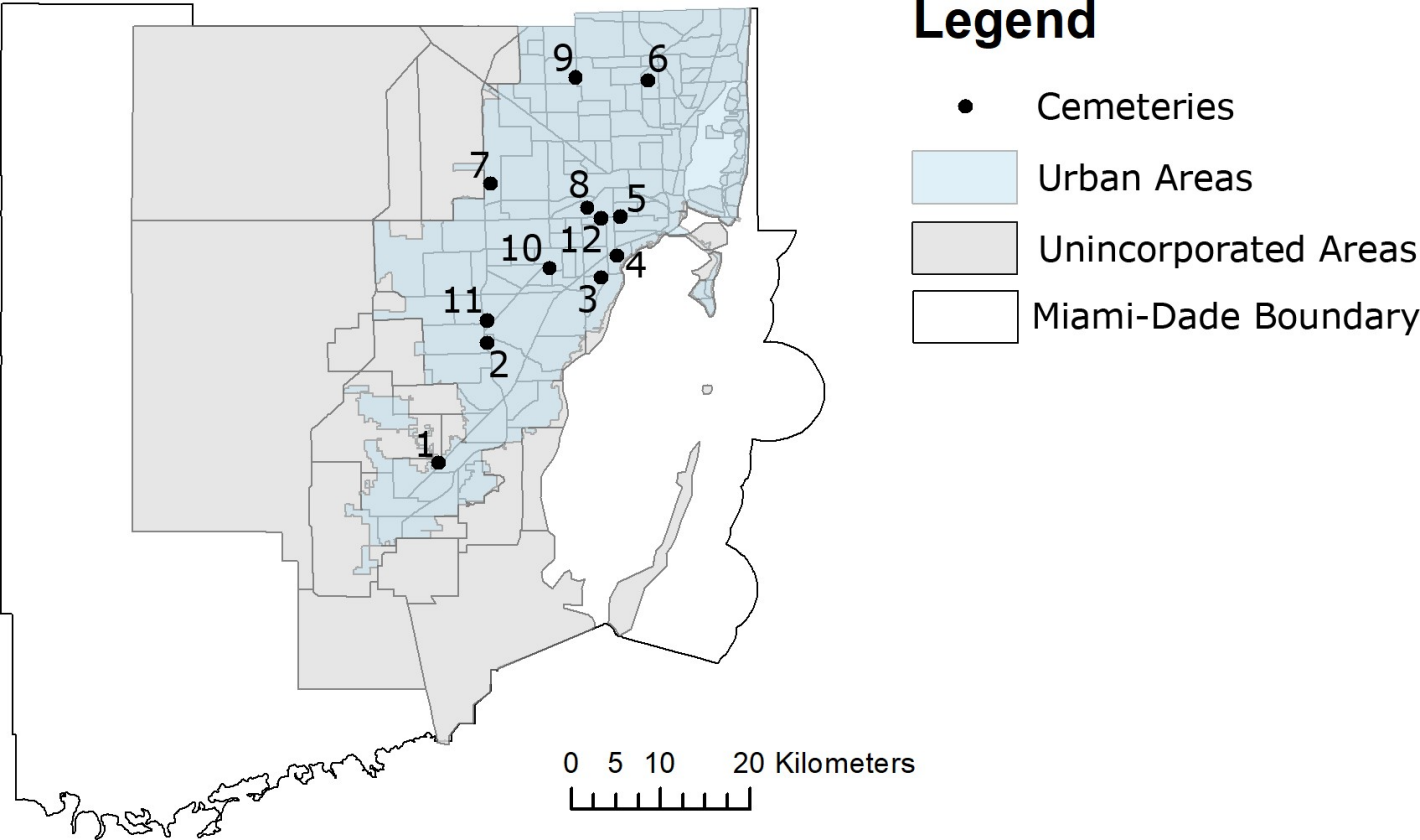
Map showing the location of the mosquito-surveyed cemeteries in Miami-Dade, Florida (latitude, 25.761681; longitude, -80.191788). Fig 1 was produced using ArcGIS 10.2 (Esri, Redlands, CA) using freely available layers from the Miami-Dade County’s Open Data Hub— https://gis-mdc.opendata.arcgis.com/.

This study used a cross-sectional design to survey the presence of vector mosquitoes in 12 cemeteries located in urbanized areas of Miami-Dade County, Florida. Aiming to provide comprehensive coverage of the cemeteries in Miami-Dade County, we selected cemeteries considering their location in different urban areas as well as their types and layouts, such as cemetery with and without tombstones or plaques and apparent or buried coffin or sarcophagus. (Fig 1).

We included in this study cemeteries with different sizes and layouts. The perimeter of the cemeteries ranged from 0.38 to 2.88 Km and the area ranged from 0.01 Km^2^ to 0.5 km^2^. All the cemeteries surveyed during this study, but cemetery 3 and 4, had similar layouts with grassed areas comprising most of the cemetery with tombstones or plaques but no apparent coffin or sarcophagus. Flower vases were present in all cemeteries, but cemetery 3.

Cemetery 3 is not currently an active burial ground and the last known burial dates in the 1940s. This area has been serving as burial grounds since the 1850s and formally became a cemetery in the 1900s. This cemetery is completely covered by vegetation and has very few ornaments other than simple tombstones on top of the graves. Cemetery 4 has a similar history. It was first used as a graveyard in the late 1850s and the land was officially purchased in the 1910s by local families. This cemetery has a unique layout with all the burials done above ground with the coffins put inside a concrete sarcophagus. All the cemeteries surveyed in this study were well maintained and relatively clean.

### Mosquito sampling and identification

Mosquitoes were collected in September and October 2019 in twelve cemeteries located in urban areas in Miami-Dade County, Florida with no on-going mosquito control. We used a standardized sampling effort for all collections. Each cemetery was surveyed once. Adult mosquitoes were collected with BG-Sentinel 2 traps (Biogents AG, Regensburg, Germany) baited with dry ice [42], for 24 hours. Two BG-Sentinel traps were used to collect adult mosquitoes at each cemetery. One trap was set near the main office and the second in a more secluded area. BG-Sentinel traps were placed in shaded areas protected from the elements to enhance the collection of mosquitoes.

Cemeteries were surveyed for immature mosquitoes for two hours or until all potential breeding sites were exhausted. Larvae and pupae were collected with manual plastic pumps (turkey basters) and stored in plastic containers (100 ml) for transport. The collected mosquitoes were transported to the Miami-Dade County Mosquito Control Laboratory. All specimens were morphologically identified to species using taxonomic keys [43]. Larvae were grown to L4 and then identified, pupae were allowed to emerge as adults and then identified, and all the adult mosquitoes collected in this study were kept at 4°C until identified.

### Data analysis

The boxplot displaying the total number of mosquitoes collected in the twelve cemeteries and the diversity profiles were created using Past software (v.3.16) [44,45]. Diversity profiles were based on the Renyi index which depends upon a parameter α (alpha). Values at α = 0 represent the total number of species for each cemetery; values at α = 1 represents an index proportional to the Shannon index (i.e., a lesser amount of importance to the presence of rare species); and values at α = 2 represent an index proportional to the Simpson index (i.e., a higher amount of importance to the presence of frequent species rather than rare species) [46,47]. The Shannon and Simpson indices [48,49], have been widely used to asses diversity variation patterns in ecological communities [50]. The Shannon index considers species abundance and communities, in which lower values indicate less diversity. On the other hand, the Simpson index estimates species dominance, values close to 0 indicate the presence of dominant species whereas values close to 1 indicate high levels of diversity [51].

A bivariate linear regression using ordinary least squares was used to estimate the association between the perimeter, area and the Normalized Difference Vegetation Index (NDVI) of the cemeteries with the relative abundance of mosquitoes. We used the 30 m Landsat 8 OLI NDVI calculated from bands 5 (near-infrared) and 4 (red) [52], which were atmospherically corrected and obtained via the US Geological Survey website, Earth Explorer (https://earthexplorer.usgs.gov/). The Landsat image was acquired on 13 January 2018. As NDVI is directly proportional to photosynthetic activity and plant canopy greenness, it provides an indicator of surface moisture and plant cover, which can affect mosquito population densities [53]. Analyses were carried out with 10,000 randomizations without replacement and a 95% confidence interval using Past software (v.3.16) [44,45].

Since this study posed less than minimal risk to participants and did not involve endangered or protected species the Institutional Review Board at the University of Miami determined that the study was exempt from institutional review board assessment (IRB Protocol Number: 20161212). Collections of mosquitoes were conducted only upon authorization.

## Results

A total of nine mosquito species were collected in the twelve cemeteries surveyed during this study. *Ae*. *aegypti*, *Ae*. *albopictus*, and *Cx*. *quinquefasciatus* were the most common and abundant species comprising 98% of all specimens collected.

*Aedes aegypti* was the most common species being found in all cemeteries, but cemetery 3, totaling 228 adults and 89 larvae and 83 pupae. *Culex quinquefasciatus* was the second most common species being found in all cemeteries but cemeteries 2 and 3, totaling 276 adults, 135 larvae and 3 pupae. *Aedes albopictus* was the third most common species being present in half of the surveyed cemeteries, totaling 51 adults, 26 larvae and 8 pupae (Table 1).

**Table 1:** Total number of mosquitoes collected at cemeteries in Miami-Dade County, Florida.

	*Aedes aegypti*		*Aedes albopictus*		*Aedes triseriatus*		*Culex coronator*		*Culex quinquefasciatus*		*Culex interrogator*		*Culex nigripalpus*		*Wyeomyia mitchelli*		*Wyeomyia vanduzeei*	
Cemetery	A	I	A	I	A	I	A	I	A	I	A	I	A	I	A	I	A	I
1	18 [20]	1	1	11					15 [25]				2			3		
2		5																
3																		
4	13 [17]				1				5 [6]								7	
5	7 [7]	22 (17)							17 [24]									
6	14 [17]		1						25 [38]									
7	17 [5]	15 (4)	1						13 [31]	1 (3)	1							
8	1 [2]	6 (9)	37 [4]	11 (8)			1		16 [27]		1				1			
9	1	(2)							5 [1]									
10	6 [1]		7	4			1		1 [5]									
11	19 [12]	40 (51)							6 [3]	134			2					
12	19 [32]								6 [7]									
Total	115 [113]	89 (83)	47 [4]	26 (8)	1		2		109 [167]	135 (3)	2		4		1	3	7	

A = adult mosquitoes, number of females and in brackets the number of males; I = immature mosquitoes; in parenthesis, the number of pupae.

**Fig 2:**
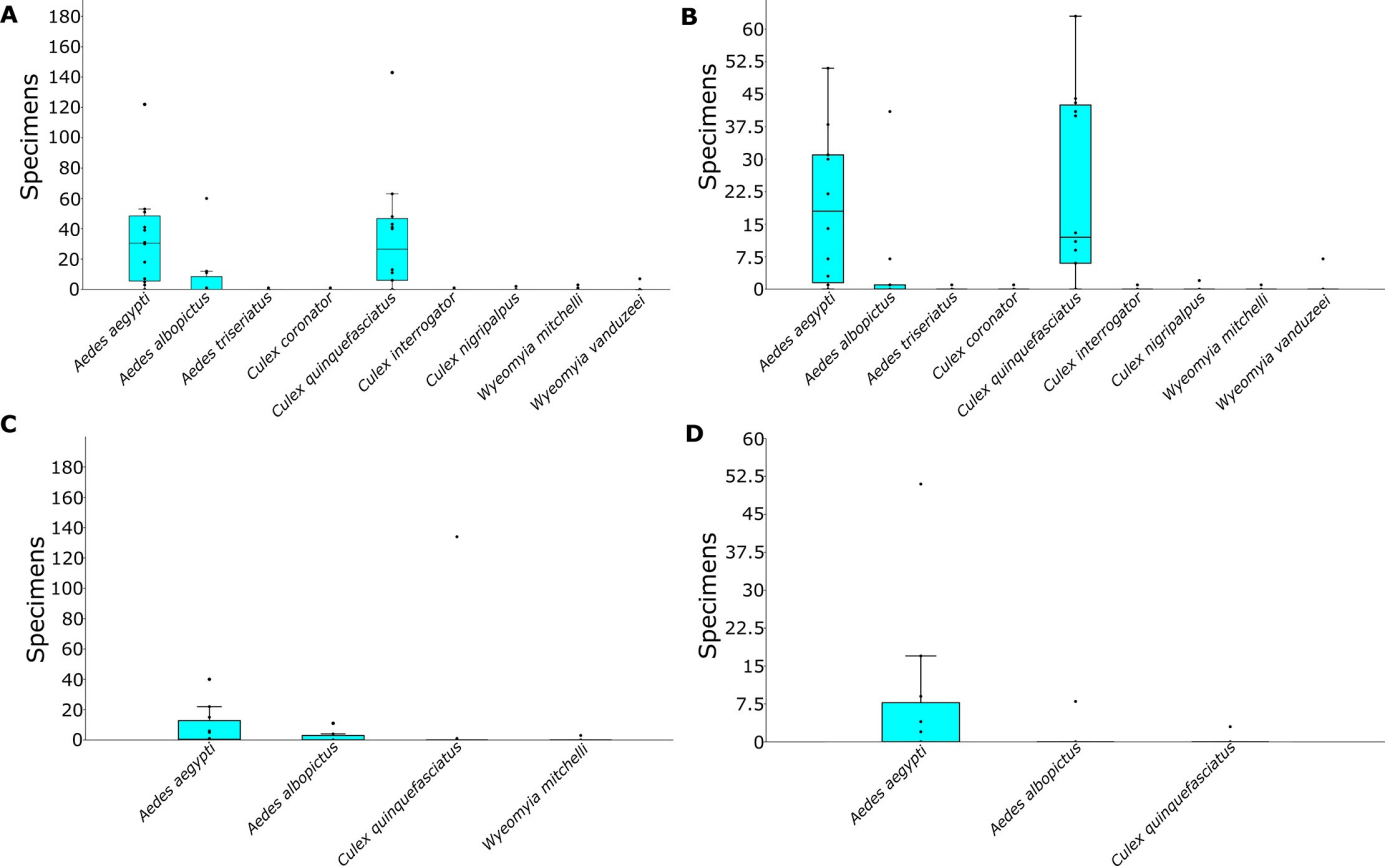
Box plot graph displaying the total number of mosquitoes collected in the twelve cemeteries surveyed in Miami-Dade County, Florida. (A) All collected mosquitoes; (B) adult mosquitoes; (C) larvae; and (D) pupae. Boxes represent the 25–75 percent quartiles; the horizontal line inside the box represents the median; the whiskers represent the largest data point less than 1.5 times the box height; and values further that limit are shown as outlier dots.

*Culex quinquefasciatus* and *Ae*. *aegypti* yielded the highest median values, 30.5 and 26.5 specimens respectively, among all specimens collected during this study (Fig 2A). A similar pattern was found for the adult mosquitoes, in which adult *Ae*. *aegypti* and *Cx*. *quinquefasciatus* mosquitoes were more abundant and yielded higher median values when compared to the other species found within cemeteries in Miami (Fig 2B). However, this pattern was not maintained in the immature mosquito population. *Aedes aegypti* was the most common species found in both the larval and pupal stage (Fig 2C and 2D).

The Bivariate Linear Regression resulted in non-significant values considering the relative abundance of mosquitoes and the perimeter, area, and NDVI of the cemeteries surveyed in this study. These results are indicative that the population dynamics of the mosquito species found in cemeteries were being driven by factors other than the increased availability of green areas such as the availability of specific conditions and resources allowing mosquito development (Tables 2 and 3).

**Table 2:** Mosquito counts and area, perimeter, and NDVI of the 12 cemeteries surveyed in Miami-Dade County, Florida.

Cemetery	Perimeter (Km)	Area (Km^2^)	Vases	NDVI	All Mosquitoes	Adults	Larvae	Pupae
1	1.54	0.15	P	0.604911	96	81	15	0
2	2.88	0.24	P	0.544393	5	0	5	0
3	0.66	0.02	A	0.813361	0	0	0	0
4	0.32	0.01	P	0.618182	49	49	0	0
5	2.46	0.28	P	0.323109	94	55	22	17
7	2.83	0.5	P	0.673231	95	95	0	0
8	2.25	0.27	P	0.492329	91	68	16	7
6	2.03	0.24	P	0.658885	124	90	17	17
9	1.96	0.23	P	0.641099	9	7	0	2
10	2.19	0.28	P	0.597605	25	21	4	0
11	2.25	0.24	P	0.44184	267	42	174	51
12	1	0.06	P	0.549094	64	64	0	0

NDVI = Normalized Difference Vegetation Index; P = Present, A = Absent.

**Table 3:** Bivariate Linear Regression for mosquitoes collected at cemeteries in Miami-Dade County, Florida.

	Perimeter			Area			NDVI		
	r	r^2^	*P*	r	r^2^	*P*	r	r^2^	*P*
All mosquitoes	0.2393	0.0572	0.4537	0.2584	0.0667	0.4173	-0.4704	0.2213	0.1227
Adults	0.1041	0.0108	0.7474	0.3292	0.1084	0.2959	-0.1812	0.0328	0.5937
Larvae	0.2053	0.0421	0.5220	0.1063	0.0113	0.7421	-0.4402	0.1938	0.1520
Pupae	0.2605	0.0678	0.4134	0.1640	0.0269	0.6104	-0.5113	0.2615	0.0892

**Fig 3:**
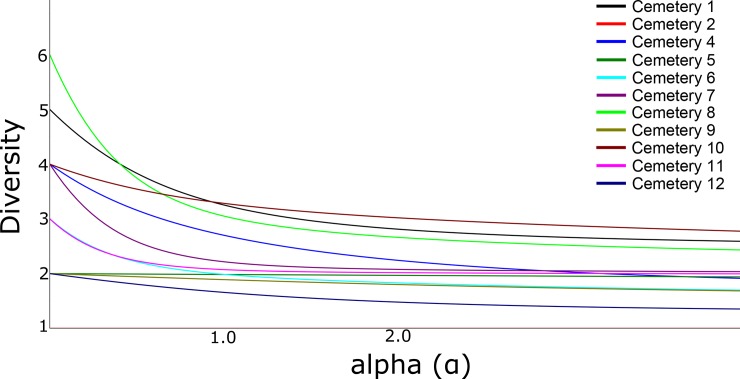
Diversity profiles considering the mosquito species collected in cemeteries in Miami-Dade County, Florida. Values at α = 0 represent the total number of species for each cemetery; values at α = 1 represent an index proportional to the Shannon index (i.e., a lesser amount of importance to the presence of rare species); and values at α = 2 represent an index proportional to the Simpson index (i.e., a higher amount of importance to the presence of frequent species rather than rare species).

The Diversity profiles analysis shows that cemetery 8 had the highest mosquito richness among the cemeteries surveyed in this study comprising 6 different species followed by cemetery 1 with 5 species, and cemeteries 4, 7, and 10 with 4 species. However, even though the species richness was relatively high considering cemeteries only comprise small areas within the urban matrix, *Ae*. *aegypti* and *Cx*. *quinquefasciatus* were the most dominant species, being present in eleven and ten out of the twelve surveyed cemeteries, respectively (Fig 3). Therefore, the analysis suggests that despite the relatively high mosquito species richness found in the cemeteries the mosquito community yielded low evenness values due to the presence of the three most dominant species, *Ae*. *aegypti*, *Cx*. *quinquefasciatus*, and *Ae*. *albopictus*.

## Discussion

Our results are indicating that vector mosquitoes are able to successfully exploit the resources available in the cemeteries allowing them to reach high densities. *Culex quinquefasciatus* was the most abundant species but it was neither as frequent nor present in its immature form as *Ae*. *aegypti*. *Aedes aegypti* was the most frequent and abundant species found in the pupal stage, which is a clear indication of mosquito production, revealing how *Ae*. *aegypti* is successfully exploiting the habitats available in the cemeteries. *Aedes albopictus* was also found in relatively high frequency and abundance in its immature form, while *Cx*. *quinquefasciatus* was found in large numbers in only one breeding site, a fishing bowl filled with rainwater placed in a shaded area.

*Aedes albopictus* is not commonly found in Miami. From August 2016 to November 2018, 150,588 adult *Ae*. *aegypti* were collected in Miami, while 11,405 *Ae*. *albopictus* were collected in the same period [6]. A similar result was found for their immature forms, from 2,488 inspections performed by Miami-Dade Mosquito Control inspectors, from April 2018 to June 2019, a total of 19,206 *Ae*. *aegypti* larvae and 2,997 pupae were collected, while only 325 *Ae*. *albopictus* larvae and 65 pupae were collected in the same period [54]. The fact that *Ae*. *albopictus* was the third most abundant species collected in the cemeteries reveals the importance of this environment to its maintenance in the urban areas of Miami-Dade County. The high epidemiological importance of both *Ae*. *aegypti* and *Ae*. *albopictus* highlights the importance of monitoring urban habitats that provide optimal conditions for mosquito development, such as in cemeteries.

Cemeteries and burial grounds are present in all urban settings around the globe. Cemeteries are complex environments and greatly vary in size, form, and shape. However, most cemeteries share similar features, such as vegetated areas with many sources of sugar and resting areas for vector mosquitoes, and widely available flower vases and ornamental plants serving as suitable aquatic habitats for vector mosquitoes. The lack of association between mosquito abundance and vegetation coverage, area, and perimeter of the cemeteries surveyed in this study shows the importance of considering local features, such as flower vases and other potential aquatic habitats for mosquitoes, for the development of effective control strategies.

Even though the role of cemeteries in the proliferation of vector mosquitoes has been studied for many years [32], not only the mosquito community composition varies from place to place but resource availability, local variation in climate and the increase in global temperatures due to global warming also have to be taken into consideration [54,55]. Constant surveillance of problematic areas for the proliferation of vector mosquitoes in urban areas is key to not only better understand how mosquito species may be exploiting these habitats but also to assess the spreading of invasive species in urban areas. *Culex coronator* has recently invaded the U.S. and was first detected in Miami-Dade County in 2008 [56]. The fact that it was found in two cemeteries highlights the importance of surveying these areas even though there is a solid body of literature on this subject.

Furthermore, as in other occupations such as in the construction workforce, cemetery workers that are responsible for landscaping and general maintenance spent a disproportional amount of time outdoors and are, therefore, subjected to vector mosquito bites being at a higher risk of being exposed to arboviruses [57–59].

In this context, cemeteries are problematic urban environments for the management and control of vector mosquitoes and should be considered key urban areas to be targeted and included in mosquito management and control strategies. The great availability of cryptic and difficult to reach aquatic habitats increases the difficulty in controlling vector mosquitoes in cemeteries. However, environmental management and removal of aquatic habitats proved to be an effective way to manage and control vector mosquitoes [30]. Furthermore, the fact that vector mosquitoes were collected all cemeteries but in cemetery 3, which had no available artificial aquatic habitat in its premises highlights the impact of anthropogenic alteration in the environment as a driver for the proliferation of vector mosquitoes in cemeteries and other problematic urban habitats.

The cross-sectional experimental design used in this study has limitations. It may have underestimated the species richness by failing to collect rare species and was unable to detect fluctuations in the mosquito community over time. However, it is appropriate to assess what are the most present and abundant species that should be targeted by mosquito control strategies.

## Conclusion

The cross-sectional mosquito survey in the cemeteries of Miami-Dade County, Florida revealed that vector mosquitoes, such as *Ae*. *aegypti*, *Ae*. *albopictus*, *and Cx*. *quinquefasciatus* are successfully exploiting the resources available in these areas being able to thrive and reach high numbers. The better understanding of how vector mosquitoes are exploiting urban habitats is vital for the development of more effective and reliable long-term mosquito management and control strategies. Even though increasing community awareness aiming to change human behavior is the ultimate goal for the development of effective mosquito management and control strategies, it is an arduous task and rarely produces immediate results. There are, however, simple practices that could greatly impact the number of available aquatic breeding habitats and as a consequence the number of vector mosquitoes. Practices as simple as drilling holes or adding larvicide to the flower vases would have a substantial effect on the availability of suitable aquatic habitats for vector mosquitoes and would help decrease the number of mosquitoes in these areas.
